# A meta-analysis of the prevalence of *Toxoplasma gondii* in animals and humans in Ethiopia

**DOI:** 10.1186/s13071-015-0901-7

**Published:** 2015-05-28

**Authors:** Endrias Zewdu Gebremedhin, Getachew Tadesse

**Affiliations:** Faculty of Agriculture and Veterinary Science, Ambo University, P.O. Box 19, Ambo, Ethiopia; College of Veterinary Medicine and Agriculture, Addis Ababa University, P.O. Box 34, Debre Zeit, Ethiopia

**Keywords:** Animals, Ethiopia, Humans, Prevalence, *Toxoplasma gondii*

## Abstract

**Background:**

Toxoplasmosis is a worldwide zoonosis. The objectives of this study were to estimate the prevalence and assess the potential risk factors of *Toxoplasma gondii* infections in animals and humans in Ethiopia by using meta-analytical methods.

**Methods:**

Published studies on *T. gondii* in animals and humans in Ethiopia were searched in Medline, Google Scholar and the lists of references of articles. Eligible studies were selected by using inclusion and exclusion criteria. The risks of within and across study biases, and the variations in prevalence estimates attributable to heterogeneities were assessed. Pooled prevalence was estimated by the DerSimonian and Laird random effects model.

**Results:**

Thirty two studies were eligible and data from 5689 animals and 5718 humans were used for quantitative syntheses. The pooled IgG seroprevalence in cats, small ruminants and humans were estimated at 87.72 % (95 % CI = 78.63, 93.28), 34.59 % (95 % CI = 21.08, 51.12) and 74.73 % (95 % CI = 61.85, 84.36), respectively. The odds of infections were higher in pregnant than in non pregnant women (OR = 3.96), in individuals that had contact with cats than those with no contact (OR = 2.53), and in urban than in rural inhabitants (OR = 2.06).

**Conclusions:**

Toxoplasmosis is highly prevalent and could be a cause of considerable reproductive wastage in small ruminants and multiple diseases in humans in Ethiopia. Public education on preventive measures could help reduce the transmission of the parasite to humans.

**Electronic supplementary material:**

The online version of this article (doi:10.1186/s13071-015-0901-7) contains supplementary material, which is available to authorized users.

## Background

*Toxoplasma gondii* is a widespread zoonotic parasite that infects all warm-blooded animals and humans [1]. Felids are its definitive hosts and excrete oocysts in their feces [2, 3], and the oocyst burden in areas where cats selectively defecate is high [4]. Animals and humans acquire infections mainly by ingesting food or water contaminated with sporulated oocysts and tissue cysts [5]. Although most infections are asymptomatic, reproductive losses in animals [6] and multiple disorders, that include cognitive impairment and fatal encephalitis in humans could come about [7, 8]. Additionally, there are reports of associations between *T. gondii* infections with schizophrenia [9, 10], bipolar disorder [11, 12], suicide [13], epilepsy [14] and traffic accidents [15, 16].

Despite *T.gondii* being an important zoonotic pathogen, there is no national survey that addressed the multiple disorders it causes in humans, its impact in animal production, its temporal and spatial distribution and the risk factors associated with the occurrence of the disease in Ethiopia. The objectives of this study were to estimate the prevalence of *T. gondii* infection in cats, food animals and humans in Ethiopia, and assess the potential risk factors of infection.

## Methods

The study was conducted according to the PRISMA guideline (Preferred Reporting Items for Systematic Reviews and Meta-Analyses) [17]. The PRISMA checklist was used to ensure inclusion of relevant information in the analysis (see Additional file 1).

### Literature search

Published studies were searched in Medline. Non-Medline indexed articles were searched in Google Scholar and the lists of references of articles. Toxoplasm* and Ethiopia were the main MeSH terms used in electronic searches. Additional searches were done by using the main MeSH terms, Boolean operators, prevalence, incidence, cats, cattle, sheep, goats, camels, pigs, chicken and humans. The last search was done on December 23, 2014. Full text articles were downloaded or obtained from the library of the school of Medicine, College of Health Sciences, Addis Ababa University, and from Dr. Jitender P. Dubey.

### Selection of studies

A study was eligible for quantitative analyses if (i) it was published in English, (ii) it was cross-sectional, (iii) the methods were coprological and/or serological [Sabin Feldman dye test (SFT), modified agglutination test (MAT), modified direct agglutination test (MDAT), direct agglutination test (DAT), enzyme linked immunosorbent assay (ELISA) and latex agglutination test (LAT)], and (iv) the sample size was greater than 35. Studies were excluded if the titles and abstracts were not relevant to the outcomes of interest or did not fulfill the eligibility criteria.

### Data extraction

From each eligible study, the following data were extracted: the first author, year of publication, year of study, location, climatic zone, altitude, study design, sample size, species, sex, age group, test methods and the number of positive samples. In addition, from studies on humans, the following were extracted: setting (hospital/non-hospital), pregnancy status (pregnant/non pregnant), HIV status (HIV positive/HIV negative), residence (urban/rural), behavior (apparently normal/abnormal), raw meat (consumer/non consumer), raw vegetable (consumer/non consumer), presence of cats in the household/contact (yes/no) and water source (pipe/others).

### Data analysis

Data on cats, food animals and humans were analyzed separately. The study level estimates were transformed to logit event estimates [18, 19] by the following formula: lp = ln [p/ (1 − p)], where lp = logit event estimate; ln = natural logarithm; p = study level estimate. The variances of the estimates were calculated by the following formula: v (lp) = 1/(np) + 1/ [n (1 − p)], where v = variance and n = sample size.

### Bias and heterogeneity analyses

The qualities of the study methods (study design and serological tests) were used to assess the within study biases. The across study bias (small study effects) was examined by funnel plots, and the statistical significance was assessed by the Egger’s regression asymmetry test [20]. The Duval and Tweedie non-parametric ‘fill and trim’ linear random method was used to calculate unbiased estimates [21]. The heterogeneities of study level estimates were assessed by Galbraith plot [22] and Cochran’s Q test. A non significant heterogeneity was accepted if the ratio of Q and the degree of freedom (Q/df) was less than one. The percentage of the variation in prevalence estimates attributable to heterogeneity was quantified by the inverse variance index (I^2^), and I^2^ values of 25 %, 50 % and 75 % were considered as low, moderate and high heterogeneity, respectively [23]. Subgroup analyses were done if the heterogeneities were moderate to high. A study was included in a subgroup analysis if the number of individuals (n) was more than 25 % of the mean of the subgroup.

### Pooling and sensitivity tests

The DerSimonian and Laird random effects model was used to pool logit event estimates [24]. Pooled logit estimates were transformed to prevalence estimates (p) by the following formula: *p* = e^lp^/ (e^lp^ + 1): where e = the base of natural logarithm. Single study omitted influence analyses were done to test the robustness of a pooled estimate, and a study was considered to have no influence if the pooled estimate without it was within the 95 % confidence limits of the overall mean. The Z test was used to test whether a pooled estimate significantly differs from zero or not. The Yates corrected Chi Square test was used to test the significance of a difference between estimates [25, 26]. Alpha was set at 0.05.

Microsoft Office Excel 2007 was used to transform study level estimates to logit event estimates, and to back transform pooled logit event estimates to prevalence estimates. Epi info^TM^ (Version 3.5.1, Center for Disease Control, CDC, USA) was used to compare groups. Stata (Version 11.1, Stata Corp, College Station, Texas) was used in all other analyses.

## Results

### Search results and eligible studies

**Fig. 1:**
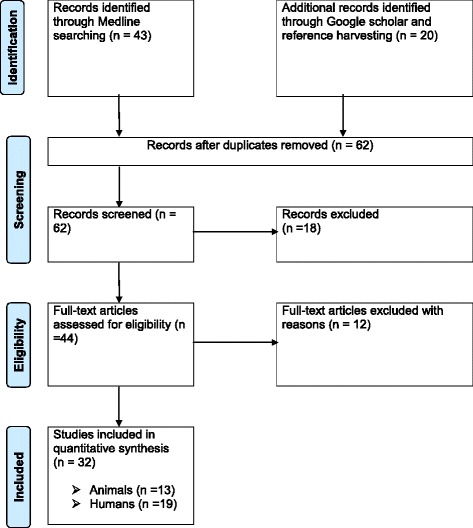
Flow diagram of the selection of eligible studies

Figure 1 shows the search results. A total of 63 studies were found of which 18 were excluded based on the titles and abstracts. Of the studies screened for eligibility, 12 were excluded due to the following reasons: one was not available; two were abstracts; the sample size was 20 in one; the data was inconsistent in one; the diagnosis was established on the basis of clinical signs in one; IHAT was used in three, and the methods were not described in three studies. A total of 32 full-text studies were used for quantitative analyses [27–58].

### Characteristics of the eligible studies

On the whole, data from 5689 animals (124 cats, 1950 sheep, 2102 goats, 726 chickens, 402 pigs and 385 camels) and 5718 humans from several regions of Ethiopia were analyzed (Table 1).

**Table 1:** Characteristics of the eligible studies

Author	Year of study	Location	Host	Method	Number	Positive (%)
Zewdu et al. [27]	2010–2011	CE	Goat	ELISA	927	183 (19.74)
Gebremedhin et al. [28]	2010–2011	CE	Sheep	ELISA	1030	357 (34.66)
Teshale [29]	2005–2006	CSE	Sheep, goat	MDAT	641	480 (74.88)
Demissie and Tilahun [30]	2000–2001	CE	Sheep, goat	MDAT	468	159 (33.97)
Negash et al. [31]	1999–2000	CE	Sheep, goat	MDAT/ELISA	174	75 (43.10)
Gebremedhin and Gizaw [32]	2013–2014	SE	Sheep, goat	ELISA	184	48 (26.09)
Gebremedhin et al. [33]	2011–2012	CE	Sheep, goat	DAT	628	111 (17.68)
Gebremedhin et al. [34]	2012–2013	CE	Camel	MAT	385	187 (48.57)
Tilahun et al. [35]	2012	CE	Chicken	MAT	125	48 (38.40)
Gebremedhin et al. [36]	2012–2013	CE	Chicken	MAT	601	183 (30.45)
Gebremedhin et al. [37]	2014	CE	Pig	DAT	402	129 (32.09)
Dubey [38]	2011	CE	Cat	MAT	36	33 (91.67)
Tiao [39]	2012	CE	Cat	MAT	48	41 (85.42)
Negash et al. [31]	1999–2000	CE	Cat	Coprology	40	5 (12.5)
Dubey [38]	2011	CE	Cat	Coprology	36	8 (22.22)
Mellbin and Vahlquist [40]	nr	WCSE	human	SFT	267	102 (38.20)
De Roever–Bonnet [41]	nr	CE	human	SFT	99	47 (47.47)
Guebre Xabier et al. [42]	1990	CSE	human	ELISA	919	673 (73.23)
Eshete et al. [43]	nr	CE	human	ELISA	94	19 (20.21)
Woldemichael et al.[44]	1995–1996	CE	human	SFT/LAT	340	268 (78.82)
Negash et al. [45]	1999–2000	CE	human	MDAT	65	39 (60.00)
Yimer et al. [46]	nr	CE	human	ELISA	279	270 (96.77)
Shibre et al. [47]^a^	2005	CE	human	ELISA	90	80 (88.89)
Shimelis et al. [48]	2007	CE	human	ELISA	330	297 (90.00)
Tedla et al. [49]^a^	2009	CE	human	ELISA	456	434 (95.18)
Zemene et al. [50]	2011	SE	human	ELISA	201	163 (81.09)
Gebremedhin et al. [51]	2010–2011	CE	human	ELISA	425	346 (81.41)
Aleme et al. [52]	2011–2012	CE	human	ELISA	150	141 (94.00)
Walle et al. [53]	nr	NE	human	ELISA	204	161 (78.92)
Muluye et al. [54]	2012–2013	NE	human	LAT	170	130 (76.47)
Tadesse et al. [55]	2012	NE	human	LAT	422	171 (40.52)
Endris et al. [56]	2010–2011	NE	human	LAT	385	341 (88.57)
Zeweld et al. [57]	2012–2013	NE	human	ELISA	651	51 (7.83)
Yohannes et al. [58]	2013	SE	human	ELISA	170	150 (88.24)

CE, Central Ethiopia; CSE, Central and Southern Ethiopia; DAT, direct agglutination test; ELISA, enzyme linked immunosorbent assay; LA, latex agglutination test; MAT, modified agglutination test; MDAT, modified direct agglutination test; MDAT/ELISA, modified direct agglutination test and enzyme linked immunosorbent assay; n, sample size; NE, Northern Ethiopia; nr, not reported; SE, Southern Ethiopia; SFT, Sabin-Feldman dye test; WCSE, Western, Central and Southern Ethiopia

^a^Data on *T.gondii* infection was collected before the trials

### Bias and heterogeneity assessment

**Fig. 2:**
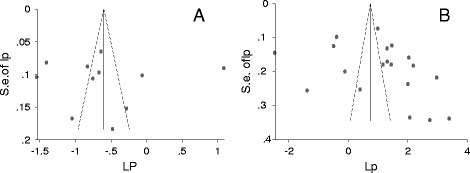
Funnel plots of the logit IgG seroprevalence estimates (lp) in food animals (**a**) and humans (**b**)

All studies on animals were cross-sectional, and the serological tests were ELISA, DAT, MDAT and MAT. All studies on humans were cross-sectional and IgG seroprevalence was examined by using ELISA, LAT, SFDT and MDAT. According to the manufacturers of the kits, the sensitivities and specificities of the tests were > = 92 %, and > = 95 %, respectively. As the sensitivity and specificity of LAT was lower than those of the other tests, a subgroup analysis of the estimates of human studies that used LAT vs. other tests did not yield a statistically significant difference (p > 0.05). The funnel plots (Fig. 2) and the bias coefficients in both food animal [b = 0.54 (95 % CI = −19.27, 20.35; p > 0.05), and human [b = 5.45 (95 % CI = −4.67, 15.57; p > 0.05) studies did not suggest the presence of bias, and no theoretical missing study was incorporated by the Duval and Tweedie non-parametric method. The percentages of the variations in prevalence estimates attributable to heterogeneities were zero, 97.6 % and 98.6 % in cat, food animal and human studies, respectively.

## Pooled prevalence estimates

### Cats

The pooled prevalence of seropositive cats was 87.72 % (95 % CI = 78.63, 93.28; p < 001), I^2^ = 0. The pooled prevalence of oocyst shedder cats was 17.51 % (95 % CI = 9.77, 29.36; p < 001), I^2^ = 19 %.

### Food animals

**Fig. 3:**
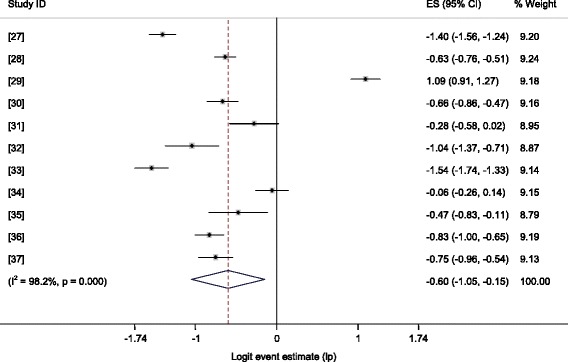
Forest plot of the logit IgG seroprevalence estimates (lp) in food animals

Figure 3 shows a forest plot of the IgG logit event estimates in food animals. The pooled estimates by potential risk factors are presented in Table 2. The overall prevalence of *T. gondii* infection was 35.5 % (95 % CI = 25.96, 46.36), and the estimates for ruminants and non-ruminants did not differ significantly (p > 0.05). In subgroup analyses, the pooled seroprevalence of *T. gondii* in small ruminants was 34.59 % (95 % CI = 21.08, 51.12; p > 0.05), I^2^ = 98.9 %, with a higher occurrence in sheep than in goats, in females than in males, and in adults than in young animals. All single study omitted estimates lie within the 95 % confidence intervals of the respective overall means of each group.

**Table 2:** Pooled estimates of T. gondii in food animals by potential risk factors

Risk factors		p (95 % CI)	Z-*p*	Q/df	Q-*p*	I^2^	*X* ^2^	Y-*p*	OR (95 % CI)
All animals	Overall	35.50 (25.96,46.36)	0.009	56.97	0.000	98.2			
	Ruminant	32.54 (22.51,44.47)	0.005	32.41	0.000	98.1	0.01	0.933	1.01 (0.87,1.16)
	Non-ruminant	32.34 (28.88,35.99)	0.000	1.5	0.223	33.4			
Small Ruminants	Overall	34.59 (21.08, 51.12)	0.067	88.69	0.000	98.9			
	Sheep	33.51 (26.06, 41.90)	0.000	10.71	0.000	90.7	10.24	0.001	1.25 (1.09,1.43)
	Goat	28.80 (11.17, 56.54)	0.129	100.39	0.000	99			
	Female	29.38 (23.06, 36.63)	0.000	9.73	0.000	89.7	23.95	0.000	1.70 (1.37,2.12)
	Male	19.67 (10.80, 33.09)	0.000	11.48	0.000	91.3			
	Adult	34.30 (24.96, 45.07)	0.005	18.79	0.000	94.7	100.50	0.000	3.06 (2.43,3.86)
	Young	14.62 (10.93, 19.26)	0.000	2.35	0.070	57.4			
	Midland ^a^	53.32 (36.12, 69.76)	0.710	16.09	0.000	93.8	45.43	0.000	2.27 (1.78,2.91)
	Highland	33.45. (29.78, 37.36)	0.000	0.28	0.597	0			
	Lowland	14.99 (8.0, 26.39)	0.000			.			

I^2^, inverse variance index; OR, odds ratio; Q, Cochran’s *X* ^2^; Y-*p,* probability of Yates test; Z-*p,* probability of Z test

^a^The comparison was between midland and highland estimates

### Humans

**Fig. 4:**
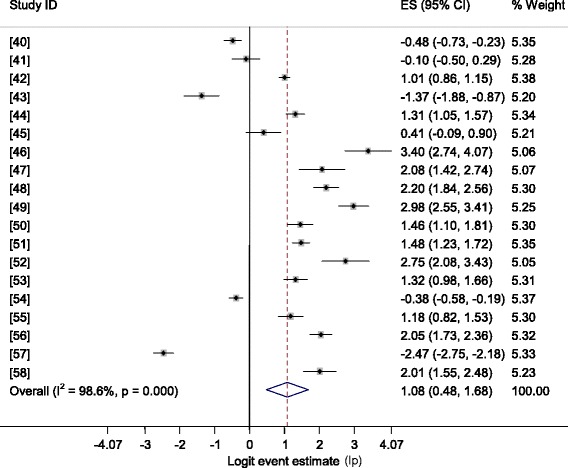
Forest plot of the logit IgG seroprevalence estimates (lp) in humans

Figure 4 shows a forest plot of the logit estimates in humans. The pooled prevalence estimates by potential risk factors are presented in Table 3. The overall pooled prevalence was 74.73 % (95 % CI = 61.85, 84.36). The pooled estimate was affected by sex, age, pregnancy, status, cat contact/ possession and residential area but not by raw meat and raw vegetable consumption habits, and source of water. The pooled seroprevalence in patients with behavioral disorders (schizophrenia/bipolar disorder) was 93.88 % (95 % CI = 81.47, 98.16; p < 001), I^2^ = 88.2 %. All single study omitted estimates lie within the 95 % confidence intervals of the respective overall means of each group.

**Table 3:** Pooled estimates of T. gondii in humans by potential risk factors

Risk factors		p (95 % CI)	Z-*p*	Q/df	Q-p	I^2^	*X* ^2^	Y-*p*	OR (95 % CI)
	Overall	74.73 (61.85,84.36)	0.000	70.4	0.000	98.6			
Sex	Male	73.54 (41.95, 91.44)	0.137	51.1	0.000	98	37.17	0.000	1.65 (1.40,1..95)
	Female	62.74 (40.08,80.91)	0.269	69.4	0.000	98.6			
Age	Adults	69.87 (47.50,85.59)	0.080	80.2	0.000	98.8	51.06	0.000	1.95 (1.62,2.36)
	Children	54.34 (24.81,81.11)	0.790	54.8	0.000	98.2			
Pregnancy	Pregnant	62.55 (29.88, 86.74)	0.462	56.8	0.000	98.2	152.83	0.000	3.96 (3.15,4.97)
	Non-pregnant	29.59 (0.75, 95.87)	0.672	197.8	0.000	99.5			
Cat	Contact	85.96 (74.12, 92.90)	0.000	13.6	0.000	92.6	64.10	0.000	2.53 (2.00,3.20)
	No contact	70.70 (53.74, 83.37)	0.018	27.4	0.000	96.4			
Residence	Urban	82.58 (73.63, 88.93)	0.000	2.7	0.000	96.3	64.04	0.000	2.06 (1.71,2.47)
	Rural	69.80 (52.40, 82.91)	0.027	19.8	0.000	94.9			
Meat	Raw C^a^	77.22 (63.85, 86.68)	0.000	18.3	0.000	94.5	0.65	0.421	1.10 (0.88,1.36)
	Raw NC^b^	75.58 (51.22.90.11)	0.040	36.0	0.000	97.2			
Vegetable	Raw C^a^	75.44 (53.00, 89.33)	0.028	31.8	0.000	96.9	1.45	0.229	1.19 (0.90,1.56)
	Raw NC^b^	72.23 (35.92, 92.34)	0.222	36.6	0.000	97.3			
Water	Non pipe	86.65 (82.17, 90.13)	0.000	0.54	0.585	0	1.36	0.243	1.30 (0.85,2.00)
	Pipe	83.45 (76.73, 88.52)	0.000	6.4	0.000	84.2			
HIV	Positive	81.06 (50.07, 94.81)	0.05	77.7	0.000	98.7	0.26	0.609	1.06 (0.87,1.29)
	Negative	80.17 (72.47, 86.13)	0.000	6.7	0.000	85.0			

I^2^, inverse variance index; OR, odds ratio; Q, Cochran’s *X* ^2^; Y-*p,* probability of Yates test; Z*-p* probability of Z test

^a^Consumers

^b^Non-consumers

## Discussion

The moderate to high inverse variance indexes of the study level estimates in food animals and humans reported here are suggestive of potential variations that could have been due to cat densities, environmental and societal factors [59, 60].

The prevalence of *T. gondii* in cats in Ethiopia was high. Ethiopian cats live outdoors, hunt, feed on scraps and garbage-thus more exposed to the parasite [3]. In a study from California, USA, the annual environmental burden per square meter was estimated to be in the range of 94 to 4671 oocysts, based on a low prevalence (0.9 %) of oocysts in cat feces [61]. Therefore, if we assume 17.51 % oocyst shedder cats in Ethiopia, and 100 million oocysts per shedder [1], the environmental burden in urban residential areas where cats abound is apparently high.

The overall pooled estimate in small ruminants was considerable (34.59 %), and the odds of infection was three times greater in adults than in young small ruminants. The higher occurrence of *T.gondii* infection in adults compared to young animals is in agreement with results of studies carried out elsewhere [62, 63]. The estimate demonstrates the potential of *T. gondii* as a cause of reproductive wastage in small ruminants, and the risk associated with the consumption of raw products derived from small ruminants [64, 65].

The substantially high prevalence of *T. gondii* infections in humans could be due to the uncontrolled movement of cats, the substandard living circumstances, and the unhygienic life styles and habits that favor the transmission of the parasite from either felids or food animals. Comparison of the present estimate with estimates for sub-Saharan African countries (SSA) is difficult because national survey reports or meta-analytical studies are scarce. However, the prevalence of *T. gondii* infection in Ethiopian women is apparently higher than estimates for most developed countries (4 % in South Korea to 11 % in USA) [60]. Therefore, assuming three million live births each year [66], and a conservative estimate of 0.1 % birth prevalence of congenital infections [67, 68], the number of congenitally infected neonates per annum could be about 3000.

*T.gondii* infection was higher in urban than in rural inhabitants and in individuals with cats in the household or had contact with cats. The cat population per unit area appears to be higher in the urban than in the rural areas because of the relatively better feed resource in the former than the latter, and as cats roam in search of food a single cat could be a source of infection to several neighboring urban households.

The high prevalence of *T.gondii* in HIV positive individuals suggests the risk of cerebral toxoplasmosis in patients that do not have access to HIV treatment. Cerebral toxoplasmosis is one of the sequels of *T.gondii* in HIV patients [69–71], and in the absence of chemo-prophylaxis against HIV, up to 40 % of patients co-infected with *T. gondii* may develop fatal *Toxoplasma* encephalitis [6]. Before the start of the highly active anti-reteroviral therapy (HAART) in Ethiopia, neural toxoplasmosis was one of the complications [72] with mortality in more than a third of the patients [72, 73], and after the initiation of HAART, immune reconstitution inflammatory syndrome (IRIS) was one of the sequels of toxoplasmosis [74–76].

To date, there have been only two studies that reported the prevalence of *T.gondii* in patients with schizophrenia/ bipolar disorders and the study level estimates were greater than 88 % [47, 49]. Meta-analytical studies have also shown higher odds of schizophrenia (OR = 2.73) [77, 78], and epilepsy (OR = 4.8) [13] in *T.gondii* seropositive than in seronegative individuals, and positive associations of *T.gondii* seroprevalence with traffic accidents [79–81], and suicide attempts and success [82] have been recorded elsewhere.

### Implications and limitations

This study demonstrates the importance of toxoplasmosis in small ruminant production and its public health impact. The control of the disease in a country where most cats are stray, and the food animal management system is mainly of an extensive type is difficult. Therefore, public education as regards the value of cooked meat and washed vegetables, and hygienic practice could reduce the transmission of the parasite and the consequences of infection in humans. Secondly, although a large scale neonatal screening program and treatment of infected neonates at a national level is not an economically viable strategy against congenital infections, initiation of such a program at least in urban areas where there are individuals who afford the medical costs could somehow reduce the impacts of the disease to a limited extent.

As the numbers of eligible studies in each stratum were small and all regions of the country have not been covered in the studies, the estimates and the predictive values of the risk factors may vary depending on factors that may involve animal, environmental and societal variables. However, the pooled estimates highlight the overall magnitude of *Toxoplasma* infections in animals and humans in Ethiopia; the heterogeneity statistics show the variability of the study level estimates and potential areas of research, and the crude odds ratios depict the relative importance of the risk factors.

## Conclusions

Toxoplasmosis is highly prevalent in Ethiopia, and could be a cause of considerable reproductive wastage in small ruminants and multiple diseases in humans. Educational programs, and serological screening and treatment of neonates when possible could help to reduce the national impacts of the disease. Further studies are needed to describe the epidemiology of the disease at a national level in Ethiopia.

## Additional file

Additional file 1:
**PRISMA Checklist.**

## References

[1] Robert-Gangneux F, Dardé M. Epidemiology of and diagnostic strategies for toxoplasmosis. Clin Microbiol Rev. 2012. doi:10.1128/CMR.05013-11. 10.1128/CMR.05013-11 PMC3346298 22491772

[2] Dabritz HA, Conrad PA (2010). Cats and *Toxoplasma*: implications for public health. Zoonoses Public Health..

[3] Dubey JP, Tiao N, Gebreyes WA, Jones JL (2012). A review of toxoplasmosis in humans and other animals in Ethiopia. Epidemiol Infect..

[4] Torrey EF, Yolken RH (2013). Toxoplasma oocysts as a public health problem. Trends Parasitol..

[5] Dubey JP (2004). Toxoplasmosis-a waterborne zoonosis. Vet Parasitol..

[6] Dubey JP (2009). Toxoplasmosis in sheep-the last 20 years. Vet Parasitol..

[7] Montoya JG, Liesenfeld O (2004). Toxoplasmosis. Lancet..

[8] Dubey JP (2010). *Toxoplasma gondii* Infections in chickens (Gallus domesticus): prevalence, clinical disease, diagnosis and public health significance. Zoonoses Public Health..

[9] Yolken RH, Bachmann S, Rouslanova I, Lillehoj E, Ford G, Torrey EF (2001). Antibodies to *Toxoplasma gondii* in Individuals with first-episode schizophrenia. Clin Infect Dis..

[10] Tanyüksel M, Uzun Ö, Araz E, Koru Ö, Babür C (2010). Possible role of toxoplasmosis in patients with first-episode schizophrenia. Turk J Med Sci..

[11] Hamdani N, Daban-Huard C, Lajnef M, Richard J-R, Delavest M, Godin O (2013). Relationship between *Toxoplasma gondii* infection and bipolar disorder in a French sample, brief report. J Affect Disord..

[12] Dogruman-Al F, Aslant S, Alcan S, Customer S, Turk S (2009). A possible relationship between *Toxoplasma gondii* and schizophrenia: a seroprevalence study. Int J Psychiatr Clin Pract..

[13] Ling VJ, Lester D, Mortensen PB, Langenberg PW, Postolache TT (2011). *Toxoplasma gondii* seropositivity and suicide rates in women. J Nerv Ment Dis..

[14] Palmer BS (2007). Meta-analysis of three case controlled studies and an ecological study into the link between cryptogenic epilepsy and chronic toxoplasmosis infection. Seizure..

[15] Yereli K, Balcioğlu IC, Ozbilgin A (2006). Is *Toxoplasma gondii* a potential risk for traffic accidents in Turkey?. Forensic Sci Int..

[16] Kocazeybek B, Oner YA, Turksoy R, Babur C, Cakan H, Sahip N (2009). Higher prevalence of toxoplasmosis in victims of traffic accidents suggest increased risk of traffic accident in Toxoplasma-infected inhabitants of Istanbul and its suburbs. Forensic Sci Int..

[17] Moher D, Liberati A, Tetzlaff J, Altman DG. The PRISMA group preferred reporting items for systematic reviews and meta-analyses: The PRISMA statement. PLoS Med. 2009. doi:10.1371/journal.pmed.1000097. PMC3090117 21603045

[18] Calvo-Muñoz I, Gómez-Conesa A, Sánchez-Meca J. Prevalence of low back pain in children and adolescents: a meta-analysis. BMC Pediatr. 2013. doi:10.1186/1471–2431–13–14. 10.1186/1471-2431-13-14 PMC3571904 23351394

[19] Hurley JC (2011). Lack of impact of selective digestive decontamination on Pseudomonas aeruginosa ventilator associated pneumonia: benchmarking the evidence base. J Antimicrob Chemother..

[20] Egger M, Davey Smith G, Schneider M, Minder C (1997). Bias in meta-analysis detected by a simple graphical test. BMJ..

[21] Duval S, Tweedie R (2000). Trim and fill: a simple funnel-plot-based method of testing and adjusting for publication bias in meta-analysis. Biometrics..

[22] Galbraith RF (1988). A note on graphical presentation of estimated odds ratios from several clinical trials. Stat Med..

[23] Higgins JP, Thompson SG (2002). Quantifying heterogeneity in a meta-analysis. Stat Med..

[24] DerSimonian R, Laird N (1986). Meta-analysis in clinical trials. Control Clin Trials..

[25] Yang Y, Li X, Zhou F, Jin Q, Gao L. Prevalence of drug-resistant tuberculosis in mainland China: systematic review and meta-analysis. PLoS One. 2011. doi:10.1371/journal.pone.0020343. 10.1371/journal.pone.0020343 PMC3108589 21674033

[26] Gao L, Zhang L, Jin Q (2009). Meta-analysis: prevalence of HIV infection and syphilis among MSM in China. Sex Transm Infect..

[27] Zewdu E, Agonafir A, Tessema TS, Tilahun G, Medhin G, Vitale M (2013). Seroepidemiological study of caprine toxoplasmosis in East and West Shewa Zones, Oromia Regional State, Central Ethiopia. Res Vet Sci.

[28] Gebremedhin EZ, Agonafir A, Tessema TS, Tilahun G, Medhin G, Vitale M, et al. Seroepidemiological study of ovine toxoplasmosis in East and West Shewa Zones of Oromia regional state, Central Ethiopia. BMC Vet Res. 2013. doi:10.1186/1746–6148–9–117. 10.1186/1746-6148-9-117 PMC3685547 23768427

[29] Teshale S, Dumetre A, Darde ML, Merga B, Dorchies P (2007). Serological survey of toxoplasmosis in Ethiopia: prevalence and risk factors. Parasite..

[30] Demissie T, Tilahun G (2002). Study on toxoplasmosis in sheep and goats in Debre Birhan and surrounding areas in Ethiopia. Bull Anim Hlth Prod Afr..

[31] Negash T, Tilahun G, Patton S, Prevot F, Dorchies PH (2004). Serological survey of toxoplasmosis in sheep and goats in Nazareth, Ethiopia. Revue Med Vet.

[32] Gebremedhin EZ, Gizaw D (2014). Seroprevalence of *Toxoplasma gondii* Infection in sheep and goats in three districts of Southern nations, nationalities and peoples’ region of Ethiopia. World Appl Sci J.

[33] Gebremedhin EZ, Abdurahaman M, Hadush T, Tessema TS. Seroprevalence and risk factors of *Toxoplasma gondii* infection in sheep and goats slaughtered for human consumption in Debre-Zeit, Central Ethiopia. BMC Res Notes. 2014. doi:10.1186/1756–0500–7–696. 10.1186/1756-0500-7-696 PMC4196015 25287190

[34] Gebremedhin EZ, Yunus HA, Tesfamaryam G, Tessema TS, Dawo F, Terefe G, et al. First report of *Toxoplasma gondii* in camels (*Camelus dromedarius*) from Ethiopia: seroepidemiology and bioassay. BMC Vet Res. 2014. doi:10.1186/s12917–014–0222–7. 10.1186/s12917-014-0222-7 PMC4189602 25266944

[35] Tilahun G, Tiao N, Ferreira LR, Oliveira S, Verma SK, Kwok OCH, et al. Seroprevalence of *Toxoplasma gondii* from free-range chicken (Gallus domesticus) from Addis Ababa, Ethiopia. J Parasitol. 2013. doi:10.1645/12–25.1. 10.1645/12-25.1 23259902

[36] Gebremedhin EZ, Tesfamaryam G, Yunus HA, Duguma R, Tilahun G, Di Marco V (2014). Seroepidemiology of *Toxoplasma gondii* infection in free range chickens (Gallus domesticus) of Central Ethiopia. Epidemiol Infect..

[37] Gebremedhin EZ, Kebeta MM, Asaye M, Ashenafi H, Di Marco V, Vitale M. First Report on Seroepidemiology of *Toxoplasma gondii* Infection in pigs in Central Ethiopia. BMC Vet Res. 2015. doi:10.1186/s12917–015–0384–y. 10.1186/s12917-015-0384-y PMC4363341 25880071

[38] Dubey JP, Darrington C, Tiao N, Ferreira LR, Choudhary S, Molla B (2013). Isolation of viable *Toxoplasma gondii* from tissues and feces of cats from Addis Ababa, Ethiopia. J Parasitol.

[39] Tiao N, Darrington C, Molla B, Saville WJA, Tilahun G, Kwok OCH (2013). An investigation into the seroprevalence of *Toxoplasma gondii*, Bartonella spp., feline immunodeficiency virus (FIV), and feline leukemia virus (FelV) cats from Addis Ababa. Epidemiol Infect.

[40] Mellbin T, Vahlquist B (1968). The antibody pattern in representative groups of Ethiopian village children. Acta Paediat Scand..

[41] De Roever-Bonnet H (1972). Toxoplasmosis in tropical Africa. Trop Geogr Med..

[42] Guebre-xabier M, Nurilign A, Gebre-Hiwot A, Hailu A, Sissay Y, Getachew E (1993). Seroepidemiological survey of *Toxoplasma gondii* infection in Ethiopia. Eth Med J..

[43] Eshete H, Tessema S, Abebe S, Abebe A (1994). Some notes on toxoplasmosis in pregnant women in Addis Ababa. Correspondence. Ethiop Med J.

[44] Woldemichael T, Fontanet AL, Sahlu T, Gills H, Messele T, de Wit TFR (1998). Evaluation of Eiken latex agglutination test for anti-toxoplasmosis antibodies and seroprevalence of toxoplasmosis infection among factory workers in Addis Ababa, Ethiopia. Trans R Soc Trop Med Hyg..

[45] Negash T, Tilahun G, Medhin G (2008). Seroprevalence of *Toxoplasma gondii* in Nazaret town, Ethiopia. East Afr J Public Health.

[46] Yimer E, Abebe P, Kassahun J, Woldemical T, Bekele A, Zewdie B (2005). Seroprevalence of human toxoplasmosis in Addis Ababa, Ethiopia. Ethiop Vet J.

[47] Shibre T, Alem A, Abdulahi A, Araya M, Beyero T, Medhin G (2010). Trimethoprim as adjuvant treatment in schizophrenia: a double-blind, randomized, placebo controlled clinical trial. Schizophr Bull..

[48] Shimelis T, Tebeje M, Tadesse E, Tegbaru B, Terefe A. Seroprevalence of latent *Toxoplasma gondii* infection among HIV-infected and HIV-uninfected people in Addis Ababa, Ethiopia: a comparative cross-sectional study. BMC Res Notes. 2009. doi:10.1186/1756–0500–2–213. 10.1186/1756-0500-2-213 PMC2770475 19852805

[49] Tedla Y, Shibre T, Ali O, Tadele G, Woldeamanuel Y, Asrat D (2011). Serum antibodies to *Toxoplasma gondii* and herpesviridae family viruses in individuals with schizophrenia and bipolar disorder: a case-control study. Ethiop Med J..

[50] Zemene E, Yewhalaw D, Abera S, Belay T, Samuel A, Zeynudin A. Seroprevalence of *Toxoplasma gondii* and associated risk factors among pregnant women in Jimma town, Southwestern Ethiopia. BMC Infect Dis. 2012. doi:10.1186/1471–2334–12–337. 10.1186/1471-2334-12-337 PMC3519766 23216887

[51] Gebremedhin EZ, Hailu A, Tessema TS, Desta K, Medhin G, Vitale M, et al. Seroepidemiology of *Toxoplasma gondii* infection in women of child-bearing age in Central Ethiopia. BMC Infect Dis. 2013. doi:10.1186/1471–2334–13–101. 10.1186/1471-2334-13-101 PMC3598201 23442946

[52] Aleme H, Tilahun G, Fekade D, Berhe N, Medhin G. Sereoprevalence of Immunoglobulin-G and of Immunoglobulin-M anti-*Toxoplasma gondii* antibodies in Human Immunodeficiency Virus Infection/Acquired Immunodeficiency Syndrome patients at Tikur Anbessa specialized hospital, Addis Ababa, Ethiopia. J Infect Dis Therapy. 2013. doi:10.4172/2332–0877.1000119.

[53] Walle F, Kebede N, Tsegaye A, Kassa T. Seroprevalence and risk factors for toxoplasmosis in HIV infected and non-infected individuals in Bahir Dar, Northwest Ethiopia. Parasit Vectors. 2013. doi:10.1186/1756–3305–6–15. 10.1186/1756-3305-6-15 PMC3556116 23324409

[54] Muluye D, Wondimeneh Y, Belyhun Y, Moges F, Endris M, Ferede G, et al. Prevalence of *Toxoplasma gondii* and associated risk factors among people living with HIV at Gondar University hospital, Northwest Ethiopia. ISRN Trop Med. 2013. http://dx.doi.org/10.1155/2013/123858.

[55] Tadese A, Mathewos B, Abebe A, Dagbew M (2013). Seroprevalence of *Toxoplasma gondii* and associated risk factors among blood donors at Gondar University hospital, Northwest Ethiopia. Int J Pharm H Care Res..

[56] Endris M, Belyhun Y, Moges F, Adefiris M, Tekeste Z, Mulu A (2014). Seroprevalence and associated risk factors of *Toxoplasma gondii* in pregnant women attending in Northwest Ethiopia. Iranian J Parasitol..

[57] Zeweld SW, Reta DH (2014). Detection of zoonotic opportunistic infections in HIV/AIDS patients in selected residential districts of Tigray region, Ethiopia. J Environ Occup Sci..

[58] Yohanes T, Debalke S, Zemene E. Latent *Toxoplasma gondii* Infection and associated risk factors among HIV-infected individuals at Arba Minch hospital, South Ethiopia. AIDS Res Treat. 2014. doi: 10.1155/2014/652941 DOI:10.1155 %2 F2014%2 F652941#pmc_ext . 10.1155/2014/652941 PMC4241326 25431660

[59] Pappas G, Roussos N, Falagas ME (2009). Toxoplasmosis snapshots: global status of Toxoplasma gondii seroprevalence and implications for pregnancy and congenital toxoplasmosis. Int J Parasitol..

[60] Flegr J, Prandota J, Sovičková M, Israili ZH. Toxoplasmosis-a global threat. Correlation of latent toxoplasmosis with specific disease burden in set of 88 countries. PLoS ONE. 2014; doi: 10.1371/journal.pone.0090203. 10.1371/journal.pone.0090203 PMC3963851 24662942

[61] Dabritz HA, Miller MA, Atwill ER, Gardner IA, Leutenegger CM, Melli AC (2007). Detection of *Toxoplasma gondii*-like oocysts in cat feces and estimates of the environmental oocyst burden. J Am Vet Med Assoc..

[62] Halos L, Thébault A, Aubert D, Thomas M, Perret C, Geers R (2010). An innovative survey underlining the significant level of contamination by *Toxoplasma gondii* of ovine meat consumed in France. Int J Parasitol..

[63] Boughattas S, Ayari K, Sa T, Aoun K, Bouratbine A. Survey of the parasite *Toxoplasma gondii* in human consumed ovine meat in Tunis City. PLoS One. 2014. doi:10.1371/journal.pone.0085044. 10.1371/journal.pone.0085044 PMC3888417 24427300

[64] Jones JL, Dargelas V, Roberts J, Press C, Remington JS, Montoya JG (2009). Risk factors for *Toxoplasma gondii* infection in the United States. Clin Infect Dis..

[65] Dubey JP, Rajendran C, Ferreira LR, Martins J, Kwok OCH, Hill DE (2011). High prevalence and genotypes of *Toxoplasma gondii* isolated from goats, from a retail meat store, destined for human consumption in the USA. Int J Parasitol..

[66] The state of the world’s midwifery, Part 4: Country profiles. Ethiopia [www.stateoftheworldsmidwifery.com]

[67] Torgerson PR, Mastroiacovo P (2013). The global burden of congenital toxoplasmosis: a systematic review. Bull World Health Organ..

[68] Flegr J (2013). How and why *Toxoplasma* makes us crazy. Trends Parasitol..

[69] Goswami RP, Goswami RP, Rahman M, Ray Y, Tripathi SK. Alternative treatment approach to cerebral toxoplasmosis in HIV/AIDS: experience from a resource poor setting. Int J STD AIDS. 2014. 10.1177/0956462414560594 25411350

[70] Akanmu AS, Osunkalu VO, Ofomah JN, Olowoselu FO (2010). Pattern of demographic risk factors in the seroprevalence of anti-*Toxoplasma gondii* antibodies in HIV infected patients at the Lagos University teaching hospital. Nig Q J Hosp Med..

[71] Addebbous A, Adarmouch L, Tali A, Laboudi M, Amine M, Aajly L (2012). IgG anti-*Toxoplasma* antibodies among asymptomatic HIV-infected patients in Marrakesh-Morocco. Acta Trop..

[72] Bane A, Yohannes AG, Fekade D (2003). Morbidity and mortality of adult patients with HIV/AIDS at Tikur Anbessa teaching hospital, Addis Ababa, Ethiopia. Ethiop Med J..

[73] BerheT, Melkamu Y, Amare A. The pattern and predictors of mortality of HIV/AIDS patients with neurologic manifestation in Ethiopia: a retrospective study. AIDS Res Therapy. 2012; doi: 10.1186/1742–6405–9–11. 10.1186/1742-6405-9-11 PMC3348055 22490062

[74] Klotz SA, Mohammed AA, Woldemichael MG, Mitku MW, Handrich M. Immune reconstitution inflammatory syndrome in a resource-poor setting. J Int Assoc Physicians in AIDS Care. 2009. doi:10.1177/1545109709332469. 10.1177/1545109709332469 19258527

[75] Huruy K, Mulu A, Mengistu G, Shewa-Amare A, Akalu A, Kassu A (2008). Immune reconstitution inflammatory syndrome among HIV/AIDS patients during highly active antiretroviral therapy in Addis Ababa, Ethiopia. Japanese J Infec Dis..

[76] Huruy K, Kassu A, Mulu A, Wondie Y. Immune restoration disease and changes in CD4+ T-cell count in HIV- infected patients during highly active antiretroviral therapy at Zewditu memorial hospital, Addis Ababa, Ethiopia. AIDS Res Therapy. 2010. doi:10.1186/1742–6405–7–46. 10.1186/1742-6405-7-46 PMC3022664 21176160

[77] Torrey EF, Bartko JJ, Lun ZR, Yolken RH (2007). Antibodies to *Toxoplasma gondii* in patients with schizophrenia: a meta-analysis. Schizophr Bull..

[78] Torrey EF, Bartko JJ, Lun ZR, Yolken RH (2012). *Toxoplasma gondii* and other risk factors for schizophrenia: an update. Schizophr Bull..

[79] Flegr J, Klose J, Novotná M, Berenreitterová M, Havlícek J. Increased incidence of traffic accidents in *Toxoplasma*-infected military drivers and protective effect RhD. BMC Infect Dis. 2009. doi:10.1186/1471–2334–9–72. 10.1186/1471-2334-9-72 PMC2692860 19470165

[80] Laura G-R, Sánchez-Orozco V, Rodríguez LR, Rodríguez S, Roig-Melo E, Sanromán RT, et al. Seroepidemiology of *Toxoplasma gondii* infection in drivers involved in road traffic accidents in the metropolitan area of Guadalajara, Jalisco, Mexico. Parasit Vectors. 2013. doi:10.1186/1756–3305–6–294. 10.1186/1756-3305-6-294 PMC3852619 24499659

[81] Flegr J, Havlícek J, Kodym P, Malý M, Zbynеk S (2002). Increased risk of traffic accidents in subjects with latent toxoplasmosis: a retrospective case-control study. BMC Infect Dis..

[82] Malhotra S, Kaur N, Kumar P, Hans C, Bhatia MS (2004). Toxoplasmosis and suicidal tendencies: is there an association?. Delhi Psychiat J..

